# Surfactant protein D attenuates acute lung and kidney injuries in pneumonia-induced sepsis through modulating apoptosis, inflammation and NF-κB signaling

**DOI:** 10.1038/s41598-018-33828-7

**Published:** 2018-10-18

**Authors:** Juan Du, Osama Abdel-Razek, Qiao Shi, Fengqi Hu, Guohua Ding, Robert N. Cooney, Guirong Wang

**Affiliations:** 1 Department of Surgery, SUNY Upstate Medical University, Syracuse, New York 13210 USA; 2 Department of Nephrology, Renmin Hospital of Wuhan University, Wuhan, 430060 Hubei Province People’s Republic of China

## Abstract

Pneumonia and sepsis are major risk factors for acute kidney injury (AKI). Patients with pneumonia and AKI are at increased risk for morbidity and mortality. Surfactant protein D (SP-D) expressed in lung and kidney plays important roles in innate immunity. However, little is known about the role of organ-specific SP-D in the sepsis. The current study uses wild type (WT), SP-D knockout (KO), and humanized SP-D transgenic (hTG, lung-specific SP-D expression) mice to study organ-specific role of SP-D in pneumonia-induced sepsis. Analyses demonstrated differential lung and kidney injury among three-type mice infected with *Pseudomonas aeruginosa*. After infection, KO mice showed higher injurious scores in both lung and kidney, and decreased renal function than WT and hTG mice. hTG mice exhibited comparable lung injury but more severe kidney injury compared to WT mice. Increased renal tubular apoptosis, NF-κB activation and proinflammatory cytokines in the kidney of KO mice were found when compared with WT and hTG mice. Furthermore, *in vitro* primary proximal tubular epithelial cells from KO mice showed more apoptosis with higher level of activated caspase-3 than those from WT mice after LPS treatment. Collectively, SP-D attenuates AKI in the sepsis by modulating renal apoptosis, inflammation and NF-κB signaling.

## Introduction

AKI is a serious complication affecting more than half of all ICU patients^1^. Clinical studies suggest pneumonia and sepsis are risk factors for developing AKI^2–4^. One multicenter and observational cohort study in the United States between November 2001 and November 2003 demonstrated that approximate one third of 1836 patients hospitalized for community-acquired pneumonia develop AKI^3^. Patients who subsequently develop AKI are at increased risk for morbidity including prolonged mechanical ventilation, hospital stay, increased costs of care and mortality compared to patients with pneumonia alone^4–6^.

Singbartl *et al*.^7^ reported *P*. *aeruginosa* pneumonia leads to AKI within 24 h, as evidenced by changes in plasma creatinine and cystatin C concentration. However, Simon found no changes in kidney structure or function in a canine model of acid aspiration-induced ALI when hemodynamics and arterial blood gas tensions are carefully controlled^8^. Based on the studies of pneumonia-induced AKI, it has been hypothesized derangements in the innate immune response, proinflammatory cytokine production, increased oxidative stress and cellular apoptosis are important in the pathogenesis of pneumonia-induced AKI through lung-kidney interactions^9,10^.

SP-D is a member of C-type lectin family, which consists of four structural domains, including a N-terminal cysteine-rich domain, a collagenous domain, a neck region and a C-terminal carbohydrate recognition domain (CRD)^11^. SP-D plays an important role in the innate host defense and the regulation of inflammatory responses in various infectious diseases^12,13^. SP-D knockout (KO) mice have shown increased susceptibility to lung infection induced by gram-negative and gram-positive bacteria, virus and fungi^14–16^. SP-D KO mice also showed increased inflammation and lung injury caused by endotoxemia^17^. By binding to pathogen-associated molecular patterns (PAMPs), SP-D facilitate the uptake and clearance of pathogens by phagocytes and epithelial cells^18^, the clearance of apoptotic cells^19^ and modulating inflammatory processes via NF-κB pathway^20,21^. We recently found that SP-A and SP-D double knockout mice showed increased *Staphylococcus aureus* pneumonia severity and intestinal injury^22^.

SP-D is predominantly expressed in lung, but is also found in extrapulmonary tissues like kidney^23–25^. Our *in vitro* study shows SP-D functions as an anti-inflammatory factor in renal tubular epithelial cells^26^. The role of SP-D in the urinary system was demonstrated by our recent studies, suggesting that SP-D together with SP-A played a protective role in the sepsis-induced AKI^27^ and urinary tract infection^28^. Additionally, SP-D could inhibit adherence of uropathogenic *Escherichia coli* to bladder epithelial cells^29^.

In this study we first generated humanized SP-D transgenic (hTG) mice with lung-specific SP-D expression, and then used wild type (WT), SP-D KO and hTG mice to investigate the mechanistic roles of organ-specific SP-D in the pneumonia-induced AKI. Furthermore, we validated the *in vivo* findings using *in vitro* primary proximal tubular epithelial cells (PTECs) isolated from WT and SP-D KO mice with LPS treatment. Our study is the first to provide evidence both pulmonary and renal SP-D play a protective role in pneumonia-induced AKI through modulating renal tubular apoptosis, NF-κB-dependent inflammation and cytokine production.

## Results

### Generation and characterization of hTG mice

**Figure 1:**
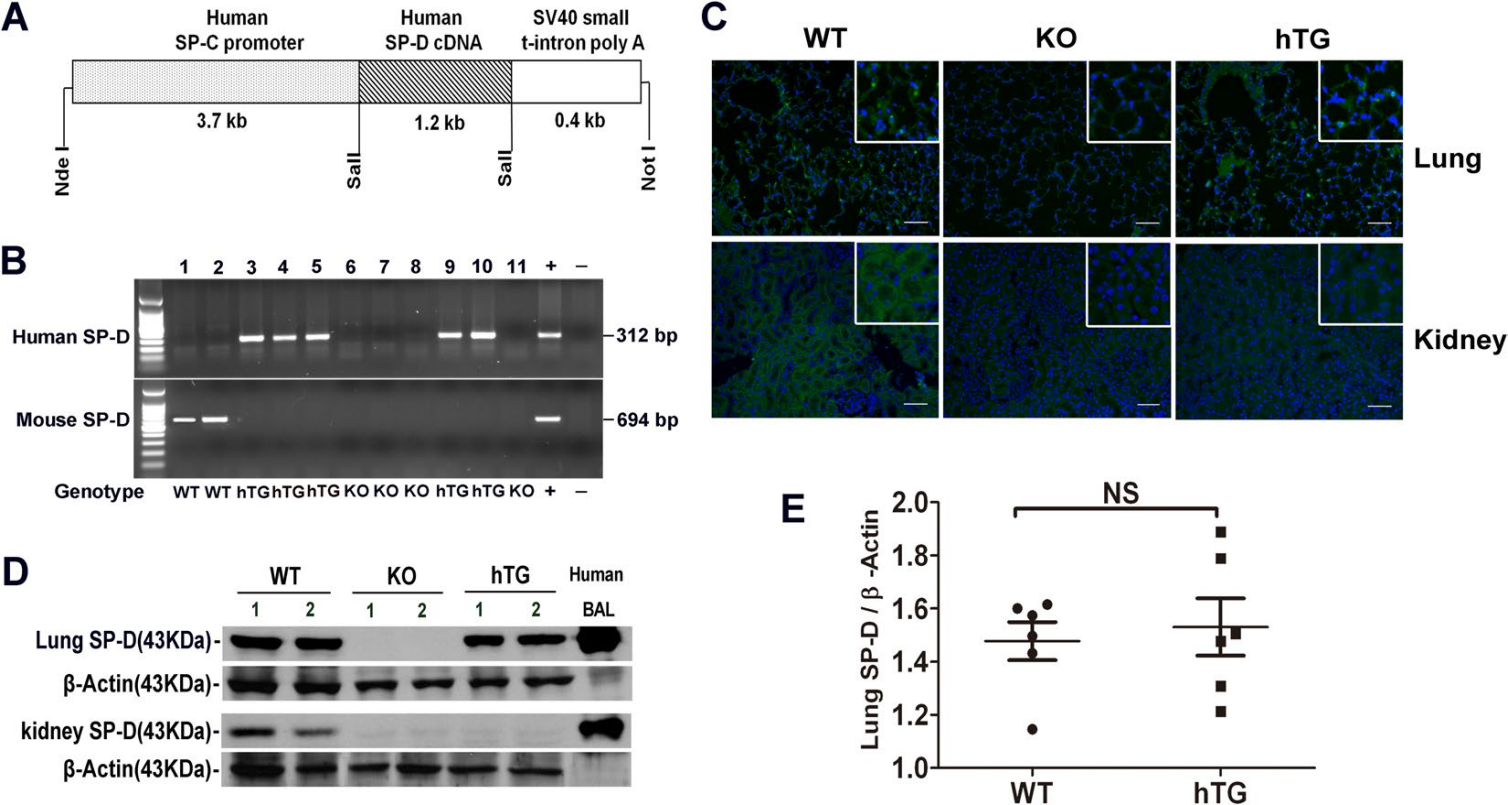
Generation of hTG mice and lung-specific hSP-D expression in hTG mice. (**A**) The diagram of recombinant DNA fragment used for the generation of hTG mice. The construct consists of a human SP-C promoter, a human SP-D cDNA and a SV40 small t-intron poly A sequence. The human SP-C promoter in the fragment drives alveolar type II cell-specific expression of hSP-D transgene. (**B**) Genotyping analysis of hTG, KO and WT mice by PCR. Recombinant plasmid was used as positive control and H_2_O was used instead of DNA template as negative control. The mice were genotyped with primers for hSP-D and mSP-D, respectively. hSP-D PCR product is 312 bp and mSP-D product is 694 bp. hTG mice carry hSP-D gene (No. 3–5, 9, and 10), WT mice have mSP-D (No. 1 and 2) and KO mice with neither hSP-D nor mSP-D (No. 6–8, and 11). (**C**) Immunofluorescent analysis for SP-D in the lung and kidney from sham WT, KO and hTG mice. SP-D expression (green color for SP-D, blue color for nuclei) was detected in both the lung and kidney of WT mice, in neither the lung nor kidney of KO mice and in the lung but not the kidney of hTG mice. (**D**) SP-D expression in the lung and kidney tissues from sham WT, KO and hTG mice by Western blotting analysis using an anti-SP-D antibody. Human BALF was used as positive control. SP-D (43 kDa) was expressed in both the lung and kidney of WT mice, in neither the lung nor kidney of KO mice, and in the lung but not kidney of hTG mice. (**E**) Quantification of SP-D expression by Western blot analysis in the lung of sham WT mice and hTG mice. The data demonstrate similar SP-D level in the lung tissues of sham WT mice and hTG mice. NS: no significance, t-test (n = 6/group).

To study the role of renal SP-D in pneumonia-induced AKI model, hTG SP-D mice with lung-specific SP-D expression were generated in this study by a DNA microinjection method with 5.3 kb DNA fragments (Fig. 1A). Five transgene positive founders were obtained. To eliminate mouse SP-D (mSP-D) gene transgenic mouse founders were crossed with SP-D KO mice for at least 8 generations before use in this study. The representative hSP-D and mSP-D genotypes of hTG, WT and KO mice were shown in Fig. 1B. hSP-D protein expression in the lung and kidney of hTG mice was analyzed by immunofluorescence (Fig. 1C) and/or Western blot (Fig. 1D) with SP-D antibody. The results showed SP-D expression in the lung of hTG mice (Fig. 1C,D). As expected, no SP-D expression was detected in the kidney (Fig. 1C,D), as well as intestine or pancreas (data not shown). Furthermore, semi-quantitative analysis indicated the comparable SP-D levels in the lung of WT and hTG mice (Fig. 1E).

### Decreased SP-D expression in the lung and kidney of infected mice

**Figure 2:**
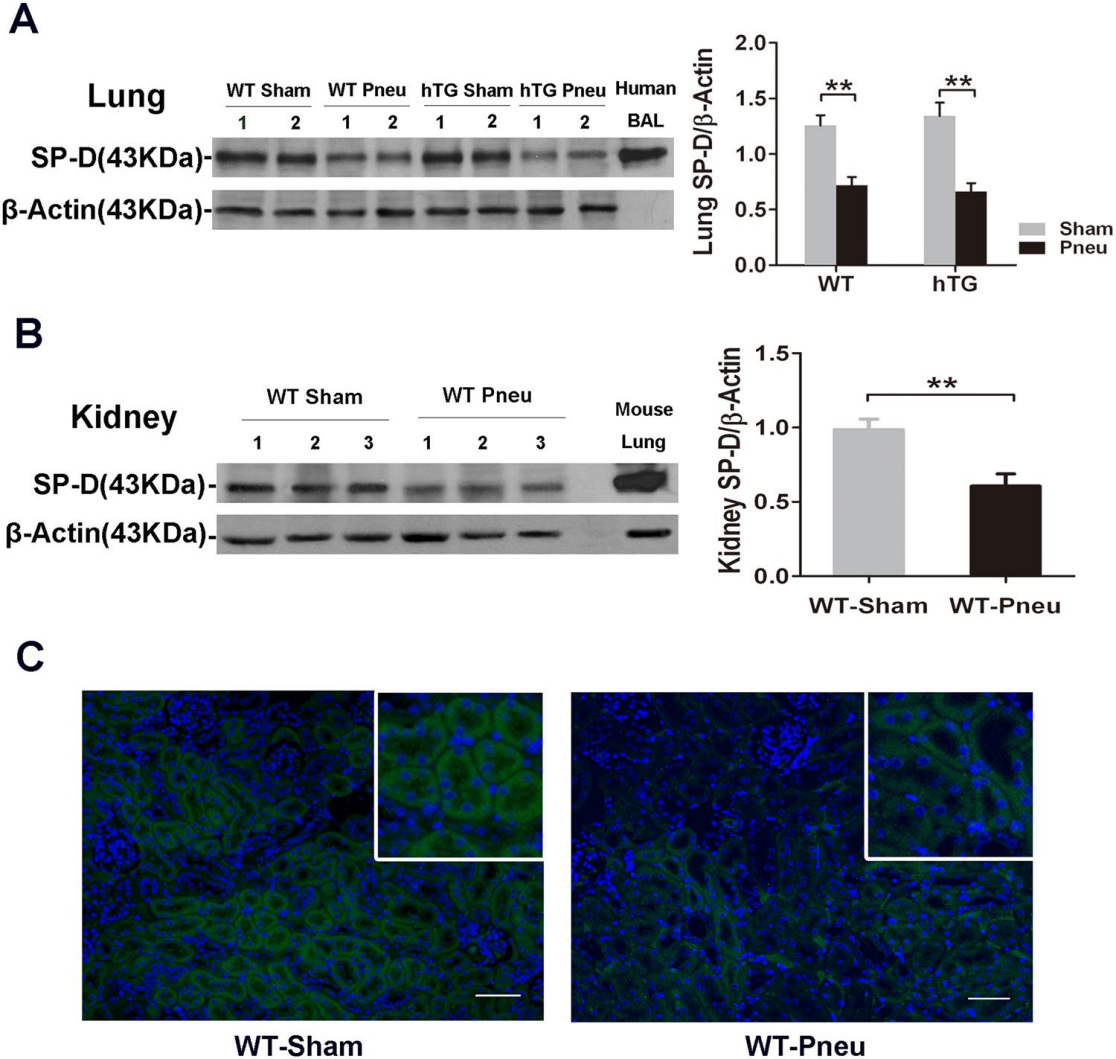
The effect of bacterial infection on the SP-D expression in the lung and kidney. (**A**) Western blot analysis of SP-D expression in the lung tissue of WT and hTG mice from sham and infected mice. SP-D expression was significantly reduced at 48 h after infection in both infected WT and hTG mice. No difference was observed between WT and hTG mice with or without infection. *P < 0.05, One-way ANOVA, Newman-Keuls multiple comparison post hoc test (n = 6/group). (**B**) Western blot analysis of SP-D expression in the kidney of sham and infected WT mice. SP-D expression was significantly lower in infected WT mice vs Sham mice. **P < 0.01, t test (n = 6/group). (**C**) Immunofluorescence staining for SP-D on representative kidney sections of WT Sham and infected mice. SP-D (green color for SP-D, blue color for nuclei) is predominantly expressed in the proximal tubular epithelial cells. SP-D expression in infected mice was remarkably reduced. Original magnification 200×. Scale bars = 100 μm. Sham = Sham infection, Pneu = Pneumonia.

To assess the effects of *P*. *aeruginosa* infection on the SP-D expression in the lung and kidney of WT and hTG mice, we performed Western blotting analysis. The results indicated that SP-D levels in the lung decreased significantly at 48 hours after infection in both WT and hTG mice compared to their respective sham group, but no difference was observed between infected WT and infected hTG groups 48 hours post-infection (Fig. 2A). In addition, reduced SP-D expression in the kidney of infected WT mice was found compared to sham mice (Fig. 2B). The immunofluorescent analysis with SP-D antibody revealed that the immunoreactivity for SP-D was primarily present in the proximal tubules of the kidney, with significant decrease in infected WT mice compared to sham mice (Fig. 2C).

### Pulmonary SP-D deficiency leads to Decreased bacterial clearance and deteriorated lung injury in pneumonia model

**Figure 3:**
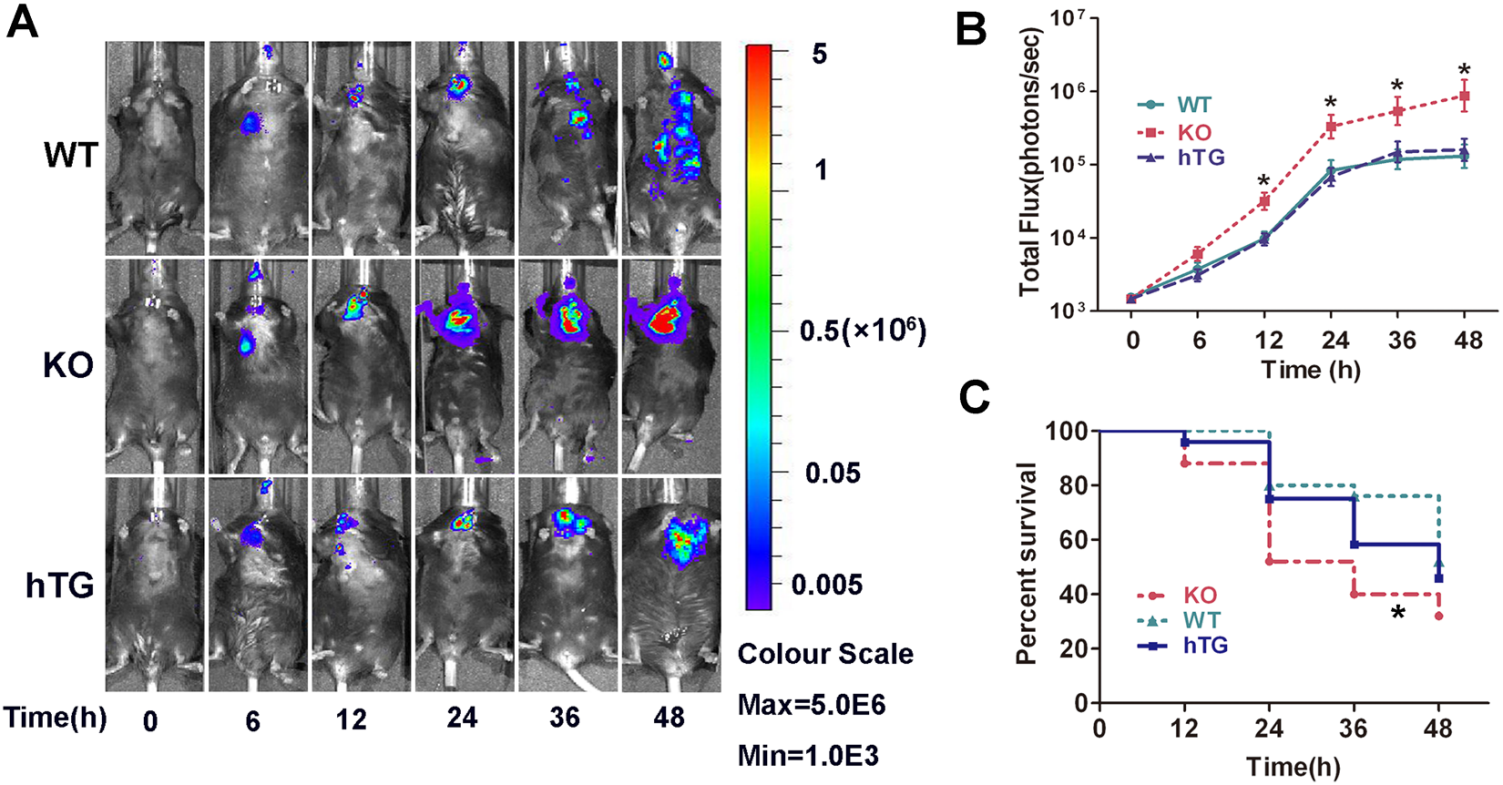
Mice lacking pulmonary SP-D are more susceptible to bacterial infection. (**A**) Representative *in vivo* bioluminescence imaging of bacteria following intratrachial inoculation of *P*. *aeruginosa* in WT, KO and hTG mice. After infection, mice were imaged at each time points as listed below. (**B**) The bioluminescence signal in three groups of infected mice progressively increased and peaked at 48 h after infection. The KO mice showed higher level of bioluminescence than WT and hTG mice at 12 h after infection and beyond, whereas no significant difference between infected WT and hTG mice. *p < 0.05 vs WT or hTG, t test (n = 6/group). (**C**) The survival curves showed lower survival rate in infected KO mice compared to infected WT, but no difference between infected hTG mice and infected WT mice. *p < 0.05 vs WT, Log-rank test (n = 25/group).

To determine the role of pulmonary SP-D in pneumonia-induced AKI, firstly we compared bacterial dynamic changes in the lungs of three types (WT, KO, and hTG) of mice at 0, 6, 12, 24, 36, 48 h after intratracheal infection of bioluminescent *P*. *aeruginosa* by *in vivo* image method (Fig. 3A). The results showed that infected KO mice exhibited significantly higher level of bioluminescence than infected WT and hTG mice at 12 h after infection and beyond, but no significant difference was observed between infected WT and hTG mice (Fig. 3B). Furthermore, a higher rate of mortality in infected KO mice was observed compared to infected WT, but no difference between infected hTG and WT mice (Fig. 3C).

**Figure 4:**
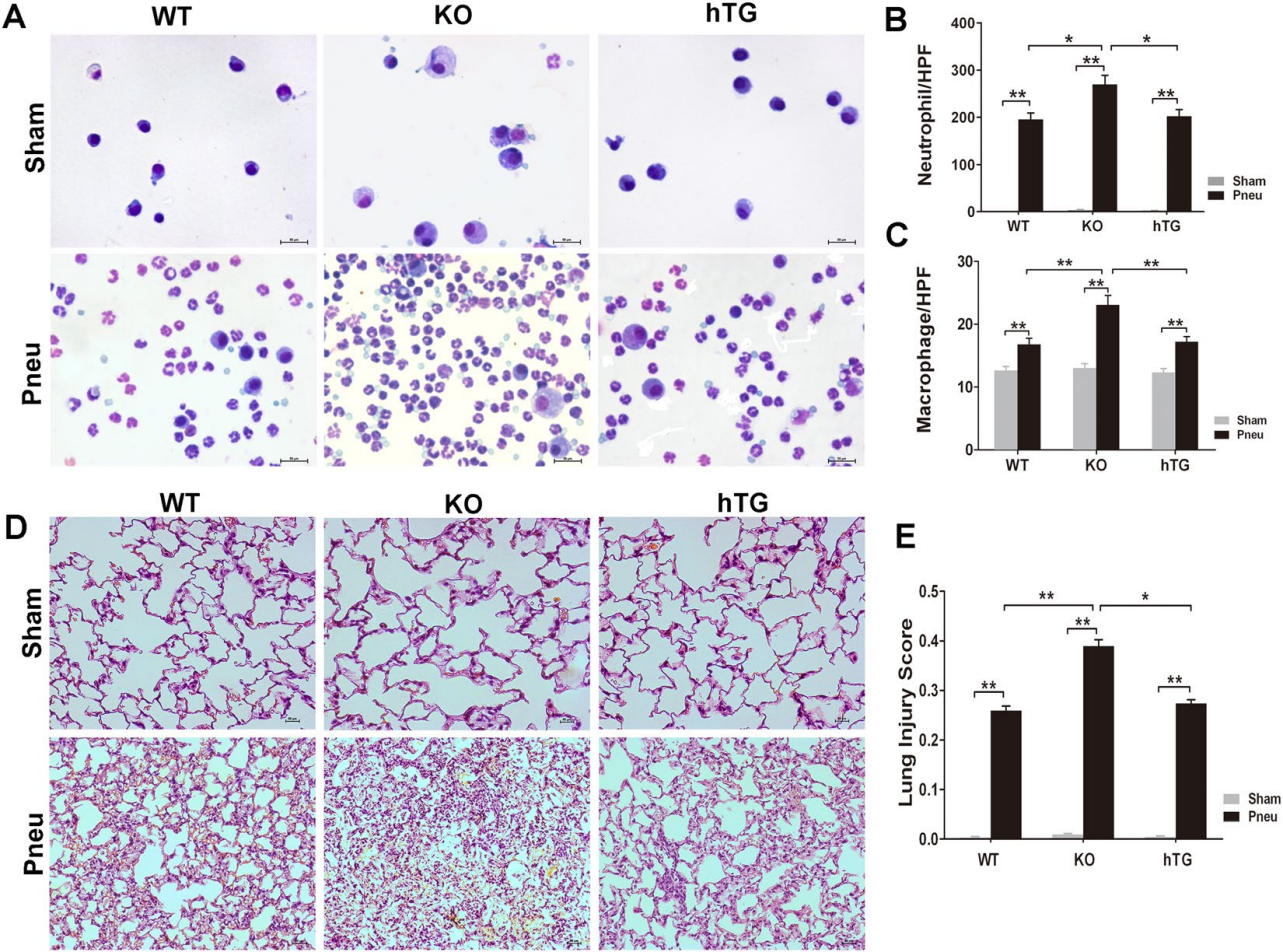
KO mice showed more severe lung injury in bacterial pneumonia compared to WT and hTG mice. (**A**) Representative BALF cytology of each group from sham and infected mice. The cell pellets of BALF were mounted on a slide by the cytospin centrifugation. The slides were stained using the Hema-3 Stain Kit. Morphologically normal macrophages with no neutrophils in sham WT mice and in hTG mice were observed. The mildly enlarged macrophages, multinucleated macrophages were observed in KO sham mice. *P*. *aeruginosa* infection causes predominant neutrophils in the BALF from three groups of infected mice. Scale bars = 100 μm. Quantification of neutrophils (**B)** and macrophages (**C**) in the BALF. Neutrophils and macrophages per slide were counted at ×400 magnification under light microscopy. There was no significant difference between infected WT and hTG mice, but the quantification was significantly higher in infected KO mice compared to infected WT and hTG mice. (**D**) Representative histological sections of lungs from each group. HE staining indicates normal lung structures in both Sham WT and hTG mice, but occasional slight enlargement of distal airspaces in Sham KO mice. Infection of *P*. *aeruginosa* causes severe lung histological damage, including diffuse inflammatory cells infiltration in alveoli and interstitial, proteinaceous debris accumulation and interstitial edema in infected mice. (**E**) Semi-quantitative histological lung injury score was assessed. There is no significant difference among sham groups. The lung injury score is significantly increased after infection compared to sham mice. There is similar lung injury score between infected WT and hTG mice, but infected KO mice showed higher injury score compared to infected WT and hTG mice. Scale bars = 50 μm; *p < 0.05, **p < 0.01, One-way ANOVA, Newman-Keuls multiple comparison post hoc test (n = 6/group). Sham = Sham infection, Pneu = Pneumonia.

As expected, alveolar macrophages were the predominant cells in the bronchoalveolar lavage fluid (BALF) of all sham groups. SP-D KO mice demonstrate accumulation of foamy alveolar macrophages and emphysema in 24-week-old aged mice^30^. Increased number and size of macrophages with occasional multinucleated macrophages as well in BALF of 8–12 weeks old KO sham mice were observed. hTG SP-D mice exhibited similar phenotypes of alveolar macrophages as WT mice, suggesting hSP-D protein expressed in the lung of hTG mice functions and rescues abnormal alveolar macrophages of KO mice (Fig. 4A). *P*. *aeruginosa* infection causes predominant neutrophils in the BALF from three groups of infected mice (Fig. 4A). The numbers of neutrophils (Fig. 4B) and macrophages (Fig. 4C) were higher in the BALF from infected KO mice compared to infected WT and hTG mice, but no significant difference was observed between infected WT and hTG mice (Fig. 4C).

Analysis of lung histopathology showed normal lung tissues in both sham WT and hTG mice but slight enlargement of distal airspaces in sham KO mice (Fig. 4D). Infection of *P*. *aeruginosa* causes severe lung damage, including diffuse inflammatory cells infiltration in alveoli and interstitial, proteinaceous debris accumulation and interstitial edema, which were similar in infected WT and hTG mice, but more severe in infected KO mice (Fig. 4D). Analysis of lung injury score demonstrated that infected WT and hTG mice had comparable injury scores, whereas infected KO mice had significantly higher score compared to infected WT and hTG mice (Fig. 4E).

### Pulmonary and/or renal SP-D deficiency renders mice more susceptible to bacterial pneumonia-induced AKI

**Figure 5:**
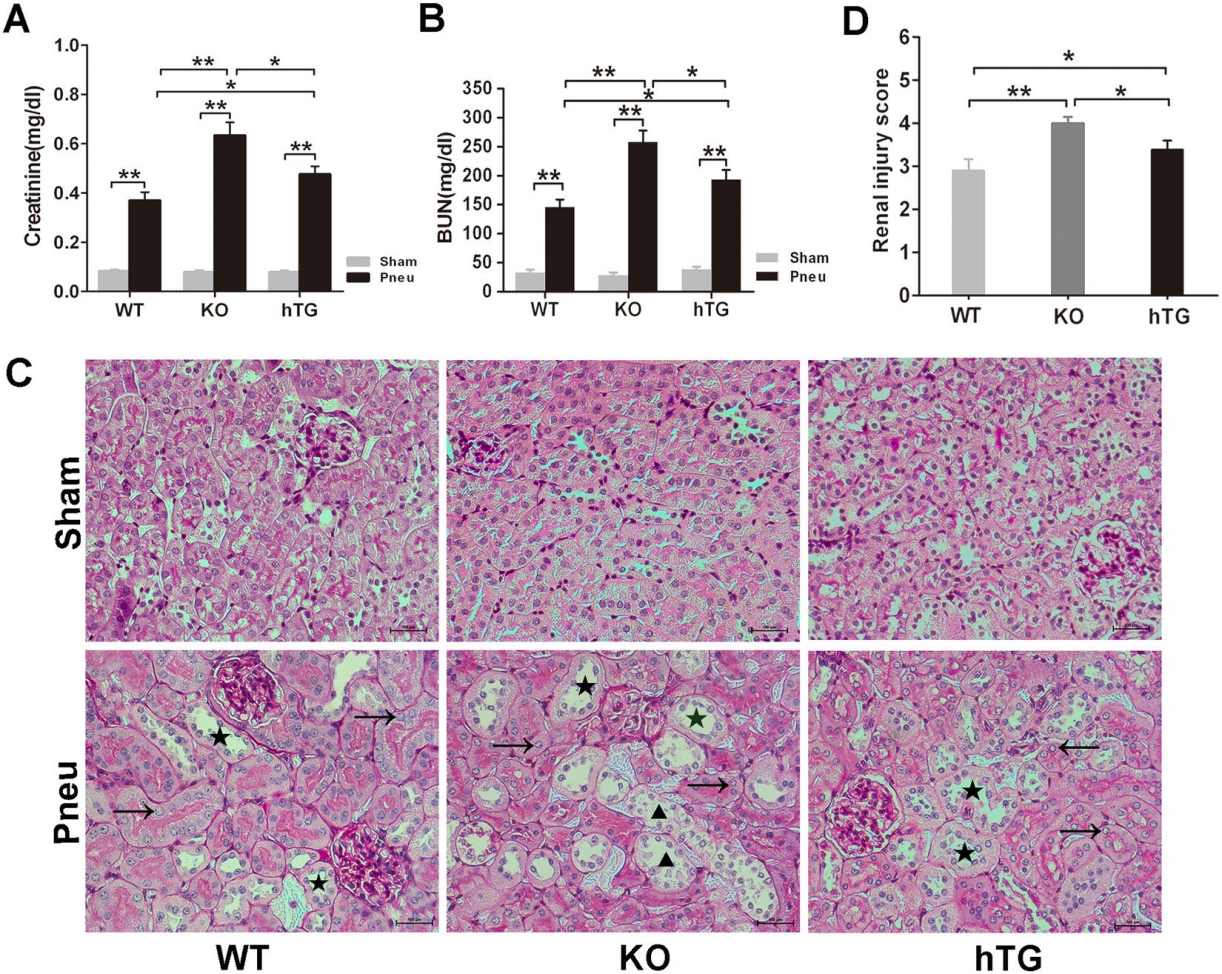
Effects of pulmonary and/or renal SP-D on bacterial pneumonia-induced AKI. The infected mice exhibited AKI by a significant elevation of Scr (**A**) and BUN (**B**). The infected hTG mice had significantly higher Scr and BUN than infected WT mice and lower Scr and BUN than infected KO mice. *p < 0.05, **p < 0.01, One-way ANOVA, Newman-Keuls multiple comparison post hoc test (n = 6/group). (**C**) Renal histology from PAS staining showed normal kidney architecture in all sham groups of mice, suggesting that SP-D deficiency in the kidney did not affect kidney development and formation of normal kidney structure. Bacterial pneumonia-induced AKI were characterized by the presence of vacuolar degeneration of tubular cells (arrows), brush border loss with tubular lumen dilatation (stars) and cast formation (triangles). Magnification 200×. Scale bars = 100 μm. (**D**) Semi-quantitative analysis demonstrated that renal injury score was significantly higher in infected KO mice compared to infected WT and hTG mice. Furthermore, when compared to infected WT mice, hTG mice had significantly higher renal injury score. *p < 0.05, **p < 0.01, t test (n = 6/group). Sham = Sham infection, Pneu = Pneumonia.

*P*. *aeruginosa* pneumonia induced AKI, as evidenced by increased levels of Creatinine (Scr) (Fig. 5A) and BUN (Fig. 5B) compared to sham groups. As expected, infected KO mice with more severe lung injury had higher levels of Scr and BUN than both infected WT and hTG mice. Interestingly, Scr and BUN levels were significantly lower in infected WT mice compared with infected hTG mice even though they exhibited similar lung injury.

To assess the degree of renal injury, kidney sections stained with PAS method were examined by two experienced renal pathologist blind to the experiments. Renal histological analysis demonstrated normal kidney architecture in all sham groups, indicating that renal SP-D deficiency did not cause spontaneous renal injury (Fig. 5C). However, dramatic pathological changes were observed in the kidney from infected mice (Fig. 5C). Infected KO and hTG mice exhibited more severe kidney damage, characterized by tubular degeneration, loss of brush border and tubular luminal cast formation when compared with infected WT mice (Fig. 5C). Accordingly, histological analyses of kidney injury indicated more intensive damage with higher renal injury score in infected KO mice compared to infected WT and hTG mice (Fig. 5D). Furthermore, infected hTG mice had higher renal injury score than infected WT mice (Fig. 5D). These findings indicated mice lacking pulmonary and/or renal SP-D were more susceptible to bacterial pneumonia-induced AKI.

### SP-D deficiency increases renal tubular cell apoptosis and caspase-3 activation

**Figure 6:**
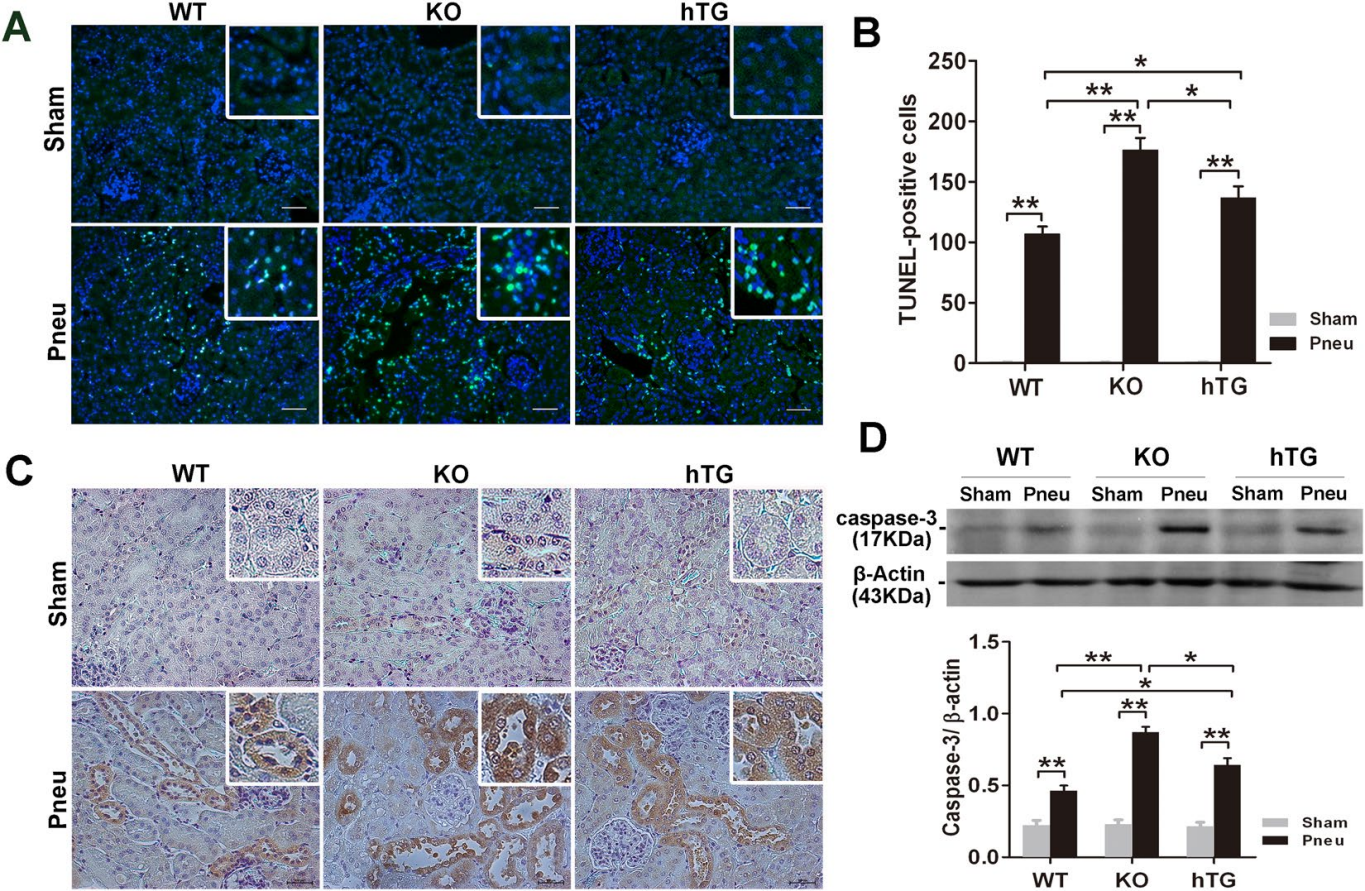
SP-D deficiency increases tubular cell apoptosis and caspase-3 activation in the kidney of bacterial pneumonia. (**A**) Representative images of TUNEL assay in the kidney of sham and infected mice 48 h after *P*. *aeruginosa* infection. The apoptotic cells showed green (blue color for nuclei). A large number of apoptotic cells are observed in the kidneys of all infected mice but not in the Sham mice. Original magnification 200×. Scale bars = 100 μm. (**B**) Apoptotic cells were quantified by counting apoptotic cells per high power field. The numbers of TUNEL-positive cells in the kidney of infected KO and hTG mice are larger than that of infected WT mice. Infected KO mice exhibit more apoptotic cells than hTG mice. *p < 0.05, **p < 0.01, One-way ANOVA, Newman-Keuls multiple comparison post hoc test (n = 6/group). (**C**) IHC assay for cleaved caspase-3 in the kidneys from Sham and infected mice. The positive staining of activated caspase-3 was characterized by cytoplasmic and perinuclear localization of brown-yellow color reaction. Caspase-3 was significantly activated and expressed on the proximal renal tubules only in infected mice but not in sham mice. Aactivated caspase-3 expression was more prominent in infected KO and hTG mice than infected WT mice. (**D**) Western blot analysis of cleaved caspase-3 expression in the kidney. Semi-quantitative analysis indicated that the level of activated caspase-3 (17 kDa) was higher in infected KO mice vs hTG or WT mice and in infected hTG vs WT mice. *p < 0.05, **p < 0.01, One-way ANOVA, Newman-Keuls multiple comparison post hoc test (n = 3/group). Sham = Sham infection, Pneu = Pneumonia.

To study the mechanisms underlying the protective role of SP-D in pneumonia/sepsis-induced AKI, we examined apoptotic cells in the kidney of infected and sham WT, KO and hTG mice. As shown in Fig. 6A, terminal deoxynucleotidyl-transsterase-mediated dUTP nick-end labeling (TUNEL) analysis revealed considerable renal tubular cell apoptosis in infected mice at 48 hours after *P*. *aeruginosa* infection, but not in sham mice. Quantitative analysis of TUNEL-positive cells indicated that the numbers of apoptosis in infected KO and hTG mice were significantly larger than that in infected WT mice (Fig. 6B). We further determined renal distribution and expression of cleaved caspase-3, an activated pro-apoptotic protein of caspase family, which plays a central role in the execution of cell apoptosis^31^. As shown in Fig. 6C, the results of immunohistochemical analysis showed increased levels of activated caspas-3 in renal proximal tubules in the kidneys of infected KO and hTG mice than infected WT mice. Furthermore, Western blot analysis demonstrated increased levels of activated caspase-3 protein (17 kDa) in infected KO and hTG mice compared to infected WT mice 48 hours after infection (Fig. 6D).

### SP-D deficiency increases renal NF-κB signaling activation and pro-inflammatory cytokine production

**Figure 7:**
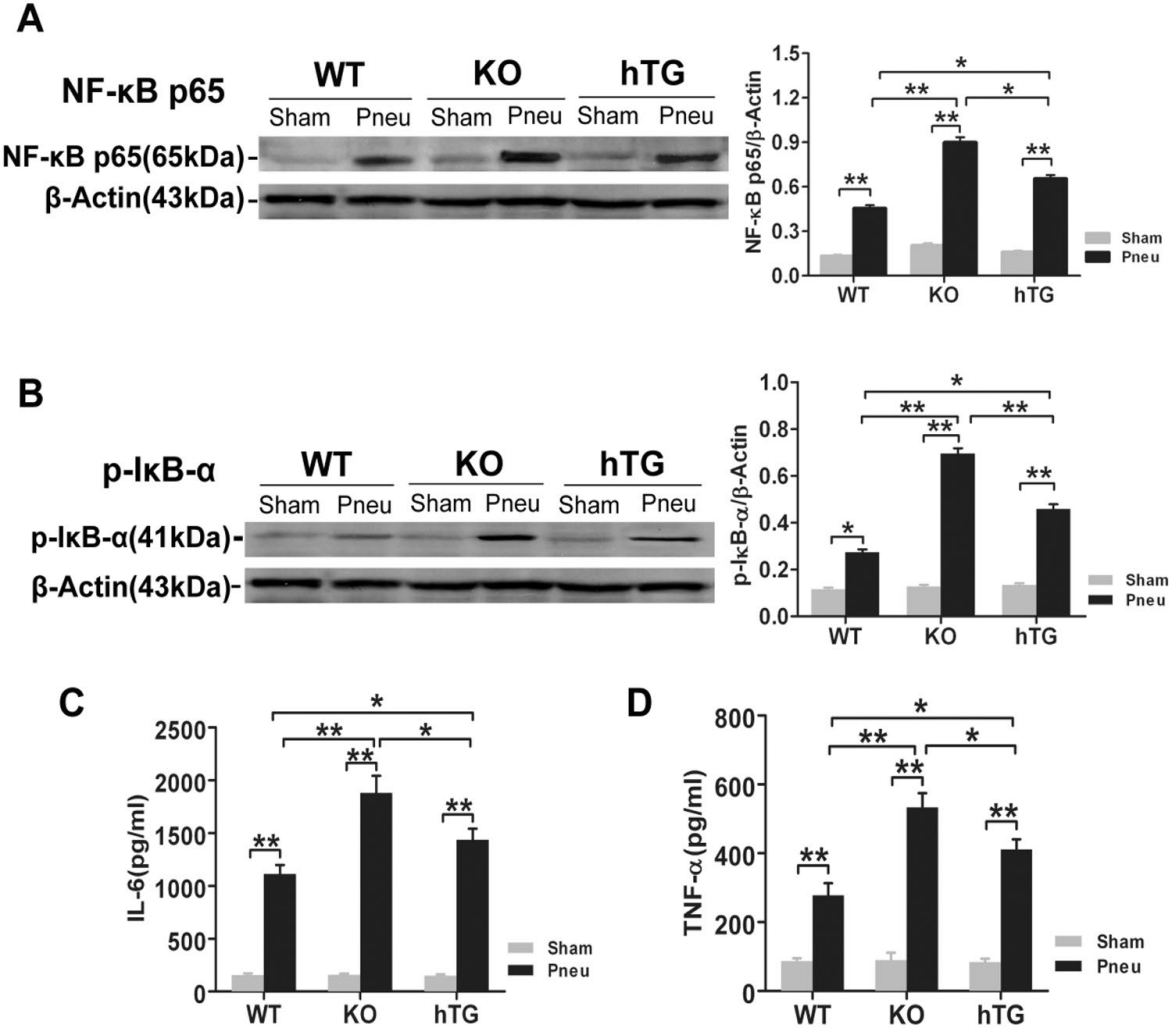
SP-D deficiency increases NF-κB activation and renal inflammation in the kidney. Western blot analysis showed increased NF-κBp65 (**A**) and p-I-κB α (**B**) expression in the kidney of infected mice compared to sham mice, indicating increased inflammatory NF-κB signaling activation the in the kidney of infected mice. Quantitative analysis indicated that the levels of NF-κBp65 and p-I-κB α in the kidney of infected mice significantly differ with an order (KO > hTG > WT), suggesting that lack of pulmonary and/or renal SP-D increases the level NF-κB activation in the kidney of bacterial pneumonia. *p < 0.05, **p < 0.01, One-way ANOVA, Newman-Keuls multiple comparison post hoc test (n = 3/group). Pro-inflammatory cytokines were determined by ELISA assay for IL-6 (**C**) and TNF-α (**D**) in the kidney of infected WT, KO and hTG mice. The results showed significantly elevated levels of IL-6 and TNF-α in all infected mice with an order (KO > hTG > WT) at 48 h after infection, suggesting inhibitory effects of SP-D in the renal inflammation of pneumonia-induced AKI. *p < 0.05, **p < 0.01, One-way ANOVA, Newman-Keuls multiple comparison post hoc test (n = 6/group). Sham = Sham infection, Pneu = Pneumonia.

NF-κB activation and pro-inflammatory cytokines are all associated with lung-kidney crosstalk in response to kidney injury^32^. Therefore, we examined renal expression of NF-κB p65 and phosphorylation of its physiologic inhibitors I-κB α (P-I-κB α) by Western blot analysis. The results showed significantly elevated NF-κB p65 and P-I-κB α in the kidneys of infected mice compared to sham mice, and there were significantly higher levels of NF-κB p65 and P-I-κB α in the kidney of infected KO mice than in infected WT and hTG mice (Fig. 7A,B). In addition, compared with infected WT mice, infected hTG mice had significant higher levels of NF-κB p65 and P-I-κB α expression (Fig. 7A,B).

We also examined the levels of pro-inflammatory cytokines IL-6 and TNF-α in kidney tissues by ELISA. At 48 hours after infection, IL-6 and TNF-α were significantly increased in three groups of mice compared to sham groups. The orders of IL-6 and TNF-α levels are KO > hTG > WT mice (Fig. 7C,D). These data further demonstrated that the lack of pulmonary and/or renal SP-D enhanced renal NF-κB activation and inflammatory cytokine expression in the kidney in pneumonia-induced AKI.

### SP-D deficiency increase *in vitro* PTEC apoptosis and expression of cleaved caspase-3 after LPS treatment

**Figure 8:**
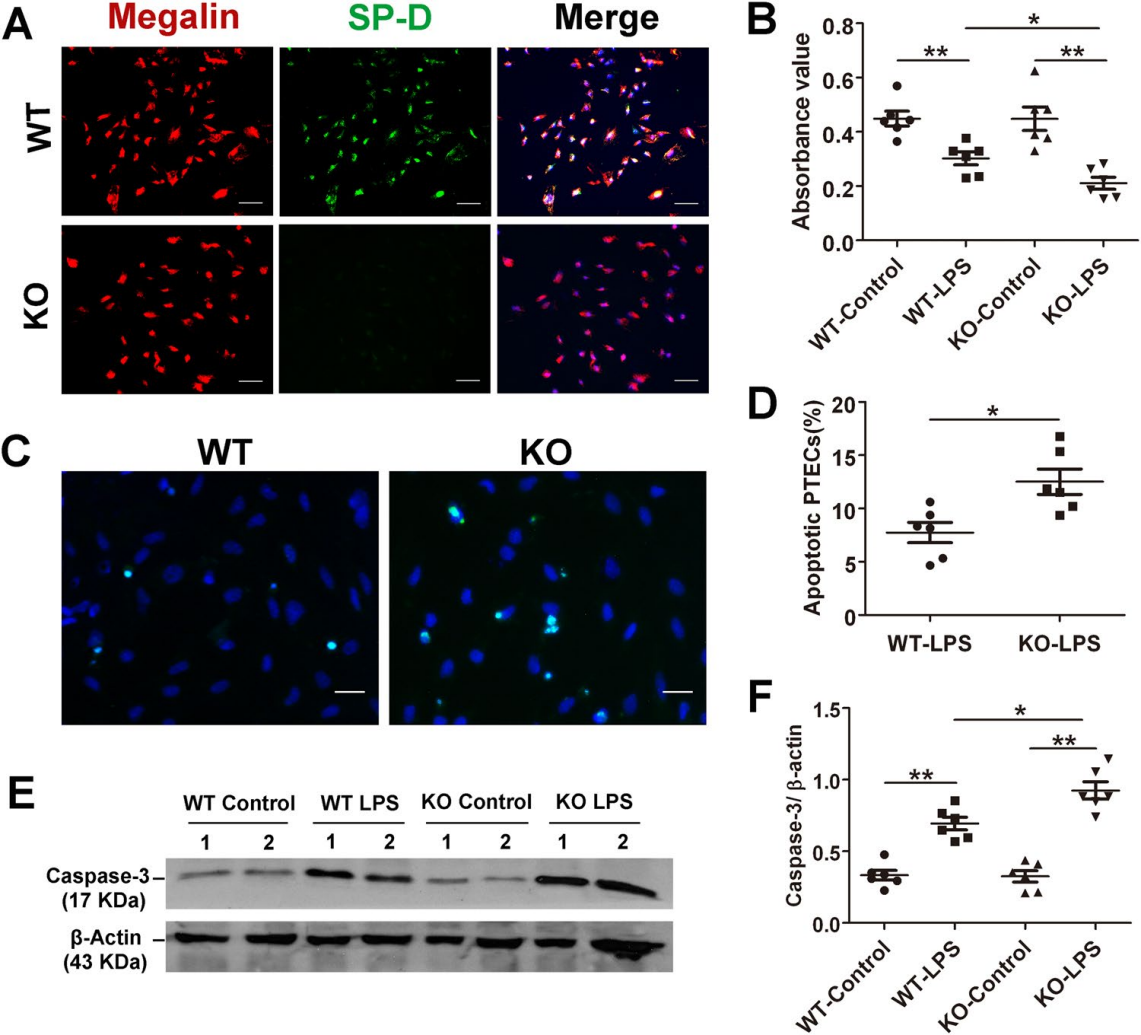
Primary proximal tubular epithelial cells (PTECs) from KO mice increase apoptosis and apoptosis-related caspase-3 expression after LPS treatment compared to those from WT mice. (**A**) PTECs from WT and KO mice were isolated and identified as described in the section of Method. More than 95% of the isolated cells from WT mice showed both megalin (red color; a biomarker of tubular epithelial cell) and SP-D positive (green color), but the cells from KO mice showed only megalin positive but SP-D negative as expected. Magnification 200×. Scale bars = 100 μm. (**B**) PTECs viability after 24 h treatment of serum-free medium with or without 10 µg/ml LPS. An exposure to LPS showed decreased cell viability compared control without LPS treatment. Treated PTECs from KO mice had lower viability than treated PTECs from WT mice. *p < 0.05, **p < 0.01, One-way ANOVA, Newman-Keuls multiple comparison post hoc test (n = 6). (**C**) Representative image of apoptotic PTECs (light green for apoptotic cells, blue color for nuclei) detected by TUNEL in WT and KO group after 24 hours treatment of 10 µg/ml LPS. Original magnification 400×. Scale bars = 100 μm. (**D**) Quantitative analysis of TUNEL positive PTECs showed that the percentage of apoptotic cells (apoptotic cells/total number of cells per ×400 field) was higher in the KO group than WT group after LPS treatment. *p < 0.05, **p < 0.01, t test (n = 6/group). Western blot analysis (**E**) and semi-quantitative analysis (**F**) of cleaved caspase-3 (17 Kda) expression in the PTECs showed increased expression of 17-KDa caspase-3 in the LPS treated cells with comparison of untreated cells, and the level of cleaved caspased-3 was higher in treated PTECs from KO mice vs. WT mice. *p < 0.05, **p < 0.01, One-way ANOVA, Newman-Keuls multiple comparison post hoc test (n = 6/group).

To study the protective effect of SP-D on tubular apoptosis, PTECs were isolated from the kidneys of WT and KO mice. The PTECs from WT and KO mice were identified by double immunofluorescence staining with megalin, a proximal tubular specific biomarker^33^ and SP-D expression. The results indicates that more than 95% of the isolated cells showed positive megalin staining (PTEC biomarker), suggesting that these isolated cells contain more than 95% of PTECs and are appropriate for following study. The double immunofluorescence staining with SP-D and megalin antibodies showed co-expressions of both proteins in PTECs from WT mice. As expected, PTECs from KO mice showed negative staining for SP-D antibody (Fig. 8A). After cells were exposed to 10 µg of LPS per ml medium in serum free medium for 24 hours, we subsequently examined the cell viability by MTT assay and apoptosis using TUNEL analysis. LPS treatment caused decreased cell viability in the PTECs from both WT and KO mice; and treated KO PTECs exhibited lower viability compared to treated WT PTECs (Fig. 8B). Consistent with the *in vivo* data, WT PTECs exhibited lower level of apoptosis compared with KO PTECs after LPS treatment (Fig. 8C,D). Furthermore, the level of cleaved caspase-3 expression was higher in treated-KO PTECs compared to treated-WT PTECs (Fig. 8E,F). These *in vitro* data support the *in vivo* findings that renal SP-D protects from tubular epithelial cells against apoptosis upon LPS treatment. In addition, we examined the effect of exogenous SP-D protein to SP-D KO PTECs after LPS treatment. The results showed that the levels of IL-6 and TNF-α in the conditioned media of LPS-treated KO PTECs in the presence of exogenous SP-D were significantly decreased compared to that in the absence of exogenous SP-D (i.e. IL-6: 335 ± 36 pg/ml vs 421 ± 45 pg/ml; TNF-α: 189 ± 25 pg/ml vs 278 + 32 pg/ml, p < 0.05, respectively), suggesting that endogenous SP-D in PTECs may interact with LPS through extracellular molecular form.

## Discussion

Pneumonia and sepsis are major risk factors inducing AKI in critically ill patients^34,35^. Until now there is only limited information about the mechanisms of ALI-induced AKI and the crosstalk between lung and kidney. Little is known about the mechanistic roles of innate immune molecule SP-D, which is expressed in both lung and kidney, in the pathogenesis of ALI-induced AKI. In the present study, we have generated and utilized hTG SP-D mice with lung-specific SP-D expression. With WT and SP-D KO and hTG SP-D mice we have explored the mechanisms underlying the role of pulmonary and renal SP-D in pneumonia-induced AKI. These findings from *in vivo* study demonstrated that lack of SP-D in lung and/or kidney promotes severe renal injury, tubular cell apoptosis, NF-κB activation and production of pro-inflammatory cytokines, which consequently results in aggravated lung-kidney crosstalk during pneumonia-induced AKI. Furthermore, *in vitro* study with isolated primary PTECs demonstrated that SP-D deficient PTECs increases cell sensitivity for apoptosis after treatment with LPS. To our knowledge, this study is the first report to demonstrate that both pulmonary and renal SP-D protects from pneumonia-induced AKI through modulating lung-kidney crosstalk and attenuating renal tubular apoptosis, NF-κB activation and pro-inflammatory cytokine production.

Many clinical and experimental studies demonstrated mechanical ventilation as an important mediator of AKI in ALI and ARDS^36,37^. Ventilation with lower tidal volumes, as opposed to traditional ventilation reduced the incidence of AKI^36–38^. Unfortunately, up to date, there are few animal studies that have examined septic ALI-induced AKI. In this study we have demonstrated that bacterial pneumonia causes AKI, as evidenced by the significantly elevated Scr and BUN levels at 48 h after bacterial infection. Histological and cellular analyses further indicate that pneumonia-induced AKI manifested remarkable acute tubular necrosis, which was characterized by bush border loss, tubular epithelial cell sloughing with focal denudation and cast formation. Singbartl *et al*.^7^ recently reported that the inhalation of *P*. *aeruginosa* only causes mild histological changes in the kidneys at 24 h, characterized by hyperemia and tubular edema. The discrepancy of these two works could be due to use of different bacterial strains or delivery methods of bacteria by the intranasal route versus intratracheal, as well as different time points of the sampling.

SP-D plays a critical role in lung surfactant homeostasis and innate immunity that protects the lung against various pathogens^12,13,39^. Our data confirmed that despite of having decreased pulmonary SP-D level 48 hrs after infection with *P*. *aeruginosa*, WT and hTG mice had increased pulmonary clearance of bacteria, decreased pulmonary inflammation and lung injury scores compared with infected KO mice. Nevertheless, hTG mice cleared bacteria as efficiently as WT mice in their lungs where they had similar levels of SP-D expression, thus resulting in similar levels of lung injury in the two types of mice. Taken together, these suggest that the decrease in clearance of *P*. *aeruginosa* and increase in lung injury observed in KO mice are due to the absence of pulmonary SP-D, which plays an important role in the innate host defense and bacteria clearance.

Infected KO mice showed a significantly higher elevation in Scr, BUN and kidney injury score than infected WT and hTG mice, which resulted from an aggravated lung-kidney crosstalk due to more severe pneumonia in mice lacking pulmonary SP-D. Survival rate in KO mice after infection was accordingly significantly decreased compared to WT mice. However, endotoxemia-induced indirect lung injury showed increased pulmonary inflammation but with a paradoxical decrease in mortality in SP-D KO mice when compared with WT mice^17^. Distinct phenotypes in direct v.s. indirect ALI models may partly explain these different observations. Direct lung injury is characterized by more severe lung epithelial injury and less severe endothelial injury, while the opposite pattern was identified in indirect lung injury^40^. Interestingly, hTG mice had more deteriorated renal function and renal histopathological changes compared with infected WT mice even though they presented similar levels of lung injury, implying the role of renal SP-D itself in attenuating lung-kidney interaction. However, in contrast to the observation in the clinical patients that a modest rise in Scr was associated with a 6.5-fold increase in the odds of death^41^, the attenuated AKI was not accompanied by significantly improved rate of mortality in infected WT mice than hTG mice, suggesting that other factors may also be involved in the morbidity and mortality of septic animals. In this work we tried to investigate the crosstalk between lung and kidney and the effect of organ-specific expressing SP-D using WT, SP-D KO and hTG SP-D mice in the pneumonia-induced sepsis. Primary injurious organ in this model is the lung due to intratracheal delivery of pathogenic bacteria. The secondary injurious organs, i.e. kidney injury, could be caused by the lack of SP-D expression but it may be influenced by the differential systemic inflammation because of differential lung injury.

Renal cell apoptosis is a major pathogenic mechanism leading to acute renal failure in AKI induced by sepsis, renal ischemic injury and nephrotoxins^42^. The involvement of renal cell apoptosis is still controversial in the pathogenesis of lung-kidney crosstalk in response to injury. Distant kidney epithelial cell apoptosis has been found in a rabbit model of acid-aspiration lung injury followed by injurious ventilatory strategies^43^. However, in a two-hit model of AKI and ALI to study kidney-lung cross-talk, only very few and weak apoptotic changes in the kidney were detected after bacterial pneumonia^7^. In the present study, we observed that renal tubular epithelial cells are highly susceptible to undergo apoptotic cell death 48 h after lung infection by markedly increased tubular TUNEL positive cells and activated caspase-3 expression.

Clearance of apoptotic cells is essential for the maintenance of tissue homeostasis and resolution of the inflammatory response^44^. Previous studies have shown that SP-D enhanced the uptake of apoptotic cells by mouse and human alveolar macrophages *in vitro* and *in vivo*^19^. SP-D KO mice have five- to tenfold higher level of apoptotic cells in their alveolar spaces and the treatment of these SP-D KO mice with an intratracheally administered human recombinant SP-D resulted in reduced levels of apoptotic cells. The pulmonary inflammation found in SP-D deficient mice might be owing to the fact that apoptotic cells cannot be cleared and they act as pro-inflammatory factors in the alveolar space^45^. In agreement with these reports in the lung, we observed that the frequency of apoptosis and expression of cleaved caspase-3 in the tubules of infected KO mice were significantly higher than those in hTG and WT mice. These findings are also consistent with our previous studies that SP-D played a protective role in sepsis-induced AKI^27^ and acute pancreatic injury^46^ by modulating renal and pancreatic apoptosis in CLP model. Therefore, these observations support the notion that SP-D deficiency in lung and/or kidney contributes to the aggravated renal tubular apoptosis, and thus causing more severe kidney injury after pulmonary infection. Recently, SP-D is found to suppress extrinsic and intrinsic apoptotic pathways of injurious T cells via reduction of caspase-8 activation^47,48^. In line with this opinion, PTECs lacking SP-D exhibited increased apoptosis, caspase-3 activation and decreased cell viability following LPS treatment.

NF-κB signaling pathway plays a critical role in the coordination of both innate and adaptive immunity and is implicated in the pathogenesis of AKI^49,50^. Inhibiting NF-κB signaling in renal tubular epithelial lead to reduce tubular injury, apoptosis, necrosis, and accumulation of interstitial inflammatory cells, which consequently results in ameliorated kidney damage after ischemic AKI^51^. Lung-kidney crosstalk is related to NF-κB dependent proinflammatory cytokines in the study of rats treated with intratracheal lipopolysaccharide instillation^32^. In this study, the significantly increased renal NF-κB p65 and p-I-κB α expression together with elevated renal TNF-α and IL-6 levels in infected mice have been observed compared to sham mice, which implicated the NF-κB-dependent proinflammatory response indeed involved in the lung-kidney interaction. In contrast, infected KO and hTG mice exhibited aggravated renal NF-κB activation and higher levels of proinflammatory cytokines release owing to the lack of lung and/or kidney SP-D expression. It seems to be explained by the ‘head or tail hypothesis’ presented by Gardai *et al*.^52^. In normal conditions when sufficient SP-D is available, SP-D binds to the signal inhibitory regulatory protein (SIRPα) of inflammatory cells via its CRD head to activate anti-inflammatory pathways. When infection occurs, the exposed collagenous tail of SP-D adheres to the calreticulin/CD91 receptor complex and initiates a pro-inflammatory response by stimulating p38 MAPK phyosphorylation and NF-κB activation. A similar dual effect was observed in the promotion or inhibition of apoptosis^53^. Therefore, the lung and kidney SP-D still plays the role of inhibiting inflammation by blocking Toll-like receptor 4 through the CRD^54^ to some extent even though they were dramatically reduced in infected WT mice, which resulted in the attenuated lung-kidney crosstalk than in KO or hTG mice.

In summary, we have generated one hTG SP-D mouse model that expresses human SP-D T allele without mouse SP-D background. We found that renal tubular necrosis/apoptosis and inflammatory responses are important mechanisms involving in bacterial pneumonia-induced AKI, in which both pulmonary and renal SP-D participates in the lung-kidney crosstalk and directly or indirectly modulate renal tubular apoptosis and its inflammatory responses. These findings indicated that both pulmonary and renal SP-D proteins are important for modulating bacterial pneumonia-induced AKI. Therefore, based on previous and present findings, SP-D may be an interesting candidate in the exogenous surfactant replacement therapy in clinical pneumonia-induced sepsis and AKI.

## Materials and Methods

### Animals

The original SP-D KO mice (C57BL/6 background) were kindly provided by Dr. Hawgood (University of California, San Francisco)^55^ and C57BL/6 WT mice were purchased from Jackson Laboratories (Bar Harbor, ME). The mice used for experiments of this study were bred and maintained in the animal core facility at SUNY Upstate Medical University. hTG SP-D mice carrying hSP-D T allele without a mouse SP-D gene background were generated in this study (see next section). There were no significant difference in the phenotype between SP-D KO, hTG SP-D and matched WT mice. Mice were housed in temperature-controlled room at 22 °C under specific pathogen-free conditions. 8–10 weeks old male and female mice were used in this study. The animal experiments were approved by the Institutional Animal Care and Use Committee of the SUNY Upstate Medical University with a protocol #380 and were performed according to the National Institutes of Health and ARRIVE guidelines on the use of laboratory animals.

### Generation and identification of hTG SP-D mice

A 5.3-kb DNA fragment (Fig. 1A) used for DNA microinjection was excised from a recombinant plasmid with restriction enzymes Nde I and Not I. The DNA fragment was consisted of a 3.7-kb human SP-C promoter, 1.2-kb human SP-D cDNA, and a 0.4-kb SV40 small t-intron poly(A) sequence. The basic 3.7-hSP-C/SV40 vector was kindly provided by Drs Stephan W. Glasser and Jeffrey A. Whitsett (Cincinnati Children’s Research Foundation, Cincinnati, OH)^56^. It was demonstrated that the transgene was specifically expressed in the lung under control of the human SP-C promoter^56,57^. The hSP-D cDNA fragment was cloned into the basic 3.7-hSP-C/SV40 vector and the recombinant construct was confirmed by DNA sequencing. The processes of recombinant DNA were performed based on the standard methods of molecular cloning^58,59^. The 5.3-kb DNA fragment was microinjected into fertilized oocytes from WT C57BL/6 mice. Human SP-D positive transgenic mice (hSP-D+, mSP-D+/+) were bred with SP-D KO mice to eliminate mouse SP-D gene background for at least five generations. All mice were genotyped using DNA from tail samples by PCR genotyping with primers for human SP-D and mouse SP-D.

### *P. aeruginosa*-induced pneumonia and sepsis model

At the initial stage of this project we performed pilot experiments with several different doses of *P*. *aeruginosa* Xen5 in both KO and WT mice to determine an appropriate dose of bacteria for this study. We observed differential degree of lung and kidney injury between infected WT and KO mice with same dose of bacterial infection. When infected KO and WT mice had similar severity of lung injury infected KO mice had more severe kidney injury, suggesting renal SP-D effect in the model. We found that a dose of 1 × 10^6^ CFU/mouse in 50 µl of bioluminescent *P*. *aeruginosa* Xen5 (a higher toxic strain) bacterial solution is appropriate in order to generate optimal bioluminescent signal in the lung detected by the *in vivo* imaging system, and to establish ALI-induced AKI with a reasonable survival rate (about 50%) at 48 h after infection. Therefore, all experiments in this study were performed through intratracheal instillation of bioluminescent *P*. *aeruginosa* Xen5 at a dose of 1 × 10^6^ CFU/50 μl/mouse to induce bacterial pneumonia^59,60^. Briefly, mice were anesthetized using intraperitoneal ketamine/xylazine (90 mg/kg ketamine, 10 mg/kg xylazine) injection. A 0.5-cm mid-line neck incision was made to expose the trachea. Bacterial solution was intratracheally inoculated into the lung of mice in pneumonia groups and 50 μl of sterile saline in sham group. At 48 h post infection, mice were anesthetized to obtain blood samples, and then sacrificed to harvest BALF, lungs and kidneys for further analyses^59^.

### *In vivo* bioluminescence imaging of *P. aeruginosa* after infection

Three groups of infected mice were monitored for a period of 48 hours after infection. Mice were anesthetized with 2.0% isoflurane. Whole body image was acquired after an exposure time of 1 min using an *In vivo* Imaging System (IVIS-200, Caliper Life Sciences, Hopkinton, MA). Bioluminescence signal of infected mice was measured at several time points, i.e. 0, 6, 12, 24, 36 and 48 h after infection. The bioluminescent signal was quantified using Living Image software, version4.1 (Caliper Life Sciences). Data are presented in physical units of radiance in photons/sec per cm^2^ per steradian^60^.

### Cytology analysis in BALF

The lung of each mouse was lavaged with 3 × 0.5 ml of sterile saline according to previously described^59,60^. The BALF was centrifuged at 250 × g for 10 min. The pellet was resuspended with 1 ml of sterile saline, and then the cells were mounted on a slide by cytospin centrifuge (Hettich ROTOFIX 32 A) at 1000 rpm for 3 min. Slides were air-dried and stained with Hema-3 Stain Kit (Fisher Scientific Company, Kalamazoo, MI) for analysis with Nikon Eclipse TE2000-U research microscope (Nikon, Melville, NY).

### Kidney function assay

Blood samples were taken through puncture of the inferior vena cava. Blood was centrifuged at 12,000 rpm for 15 min at 4 °C to obtain serum. Serum creatinine (Scr) was determined using a commercial assay kit (Thermo Scientific, Middletown, VA). Blood urea nitrogen (BUN) was analyzed using a Roche Diagnostic analyzer (Roche, Indianapolis, IN)^27^.

### Histopathological analysis

Lungs or kidneys were inflation-fixed by means of tracheal instillation of 0.5 ml of neutral formalin. Fixed lung and kidney tissues were embedded in paraffinas as described previously^27,60^. About 5μm sections of lung tissues and 3μm sections of kidney tissues were cut and stained with Hematoxylin and Eosin (H&E) and periodic acid-Schiff (PAS), respectively. Histopathology was evaluated by two independent pathologists. Lung injury was evaluated using a 0–2 scale^61^. Kidney injury was semi-quantified by counting the percent of tubules that displayed cell necrosis, loss of brush border, cast formation, and tubule dilatation as previously described: 0 = none, 1 = <10%, 2 = 11–25%, 3 = 26–45%, 4 = 46–75%, and 5 = >76%, as described previously^62^.

### Immunohistochemistry

Analysis of immunohistochemistry (IHC) was performed as described previously^46^. The paraffin-embedded kidney sections were de-waxed and rehydrated. Epitope retrieval was carried out by boiling in 10 mM citrate (pH 6.0) for 15 min. The slides were immersed into 3% H_2_O_2_ for 30 min to block endogenous peroxidase activity. After incubating with 10% goat serum for 60 min at room temperature, the sections were added with anti-cleaved caspase-3 primary antibody (#9661, Cell Signaling Technology) at 4 °C overnight. Subsequently, a HRP-conjugated secondary antibody (#170-6515, Bio-Rad, Hercules, CA) at 1:200 dilution was applied for incubation in a humidified chamber at room temperature for 60 min. DAB was used as a chromogenic substrate to develop color in the section for 1 min. Finally, the sections were counterstained for 1 min with hematoxylin. Slides were viewed by a Nikon Eclipse TE2000-U research microscope (Nikon, Melville, NY).

### Immunofluorescence

For lung and kidney tissues, the paraffin-embedded sections were deparaffinized and immersed in 0.2% Triton X-100 for 45 min. After blocking with 10% donkey serum (ab7475, Abcam Inc, Cambridge, MA) in PBS for 1 h, slides were immunostained with rabbi anti-SP-D antibody (gift from Dr. Wright, Duke University Medical Center, Durham, NC, USA)^28^. For identification of primary proximal tubular cells, after fixing with 4% paraformaldehyde, the cells planted onto glass coverslips were double stained with anti-SP-D and anti-megalin antibody (sc-16478, Santa Cruz Biotechnology, Dallas, Texas) to confirm the species of cultured cells and to determine the purity of proximal tubular epithelial cells. For fluorescence visualization of primary antibodies, slides were stained with Alexa 488 (ab150073, Abcam Inc, Cambridge, MA) and/or Alexa 596-conjugated secondary antibodies (A11058, Life Technologies, Eugene, OR) for 1 h at room temperature.

### Apoptotic cell detection by TUNEL assay

Formalin-fixed, paraffin-embedded, 5μm-thick renal tissue sections or 4% paraformaldehyde-fixed primary proximal tubular cells were treated with Click-iT Plus TUNEL Assay Kit (C10617, Molecular Probes, Eugene, OR) according to the instructions of the manufacturer. Slides were mounted by fluoroshield mounting medium with DAPI (ab104139, Abcam Inc, Cambridge, MA) to visualize cell nuclei. Apoptotic cells were quantified at each high power field (×400 magnification) by counting the number of TUNEL-positive cells on 10 randomly chosen view fields.

### Western blotting analysis

Western blot analysis was performed according to description in our previous works^27,46,59^. In brief, lung and kidney tissues were homogenized in RIPA buffer containing a cocktail of protease and phosphatase inhibitors (Roche, Indianapolis, IN) as well as aprotinin (MP Biomedicals, LLC, Illkirch, France). After centrifugation at 13000 rpm for 10 min, the supernatants were harvested for Western blot analysis. Total protein concentrations of lung and kidney sample were determined by using the BCA protein assay kit (Thermo Scientific, Rockford, IL). Thirty microgram of total proteins for each sample was resolved by reducing, electrophoresed on 12% SDS-polyacrylamide gel, and then transferred onto PVDF membranes (Bio-Rad, Hercules, USA). The blot was blocked in Tris-buffered saline containing 5% non-fat milk for 1 h, and was subsequently incubated with a primary antibody against SP-D, or NF-κB p65 (sc-109, Santa Cruz Biotechnology, Dallas, Texas), or phosphorylated I-κB α (sc-101713, Santa Cruz Biotech, Dallas, Texas), or cleaved caspase-3 (#9661, Cell Signaling Technology) at 4 °C overnight. A β-actin antibody (sc-130657, Santa Cruz Biotech, Dallas, Texas) was used as internal control. Thereafter the membranes were incubated with a HRP-conjugated secondary antibody (Bio-Rad, Hercules, CA). The blots were detected using Pierce ECL Western Blotting Substrate (Thermo Scientific, Rockford, IL) and exposed to X-ray film (Pierce Biochemicals, FL). The relative expression of protein was quantified by ImageJ software version 1.48 (Wayne Rasband, NIH, Bethesda, MA). For determination of SP-D, human BALF or proteins from lung tissue in sham mouse were used as positive control. In some experiments, blots were stripped to remove antibody by incubation in 2% SDS, 0.06M Tris/HCl (pH 7.0), and 0.72 M 2-mercaptoethanol for 30 min at 50 °C, and then reprobed with a second primary antibody^63^.

### ELISA assay for cytokines

The levels of IL-6 and TNF-α in renal homogenate or conditioned media from cell culture were assayed using commercially available mouse ELISA kits according to the manufacturer’s instructions (KMC0061 and KMC3011, Life Technologies, Frederick, MD).

### Primary Proximal Tubular Epithelial cell (PTECs) isolation and culture

Primary PTECs from WT and KO mice were isolated under sterile conditions using previously described methods with a few modifications^64,65^. Renal cortices were dissected visually in ice-cold HBSS and sliced into small pieces. The fragments were digested in PBS buffer containing 1 mg/ml type I collagenase (Worthington, Lakewood, NJ) and 125 μg/ml defined trypsin inhibitor (Gibco) at 37 °C for 30 min. The supernatant was sieved through two nylon sieves (pore size 200 um and 70 um). Tubular fragments caught by the 70-μm sieve were flushed in the reverse direction with PBS and centrifuged for 5 min at 200 × g. Thereafter the pellet was resuspended in DMEM/F12 medium, overlayed on an OptiPrep^TM^ density gradient solution (Sigma-Aldrich) and centrifuged for 20 min at room temperature 800 × g^66^. Highly purified proximal tubules in the fraction between 1.05 and 1.076 g/ml were collected^67^, washed twice in ice cold PBS and maintained in DMEM/F12 medium supplemented with 5% FBS, 1× MEM nonessential amino acids, 1 × insulin-transferin-selenium, 100 IU/ml penicillin and 100 ug/ml streptomycin. The plate was incubated in a humidified incubator under 5% CO_2_ at 37 °C. The medium was changed every other day until 90% of cell cultures were organized as a confluent monolayer.

### PTECs treatment with LPS

PTECs isolated from WT and KO mice were treated with 10 μg/ml LPS or left untreated in serum-free medium for 24 hours. Thereafter, medium and cells were harvested for further analysis. Optimal concentration of LPS and length of treatment were determined by pilot studies. In an additional experiment, PTECs from SP-D KO mice were treated with 10 μg/ml LPS in the presence or absence of exogenous SP-D protein (10 µg/ml) for 24 hours and then the conditioned media from cultured cells were harvested and used for cytokine analysis.

### MTT assay

Cell viability was assessed by the MTT assay following the manufacturer’s instruction (v13154, Molecular Probes, Eugene, OR). In brief, cells were grown in 96-well cell culture plates at a density of 5 × 10^4^ cells/well for 48 h. After 24 h of desired treatments, remove the medium and replace it with 100 μl of fresh culture medium. Then add 10 μl of 5 mg/ml MTT dissolved in PBS to each well. After incubation at 37 °C for 2 h, add 100 μl of the SDS-HCl solution (1 g of SDS in 10 ml of 0.01 M HCl) and incubate at 37 °C for 4 h. The absorbance was read at 570 nm.

### Statistical analysis

All data were presented as mean ± SEM. Statistical analysis of the data was made using GraphPad Prism software (version 5.0). Comparison among/between groups was performed using One-way ANOVA or t-test. Animal survival was performed by the Kaplan-Meier survival analysis. For all comparisons, p < 0.05 was considered statistically significant.

## References

[1] Hoste EA (2015). Epidemiology of acute kidney injury in critically ill patients: the multinational AKI-EPI study. Intensive Care Med.

[2] Mehta RL, Pascual MT, Gruta CG, Zhuang S, Chertow GM (2002). Refining predictive models in critically ill patients with acute renal failure. J Am Soc Nephrol.

[3] Murugan R (2010). Acute kidney injury in non-severe pneumonia is associated with an increased immune response and lower survival. Kidney Int.

[4] Darmon M (2014). Acute respiratory distress syndrome and risk of AKI among critically ill patients. Clin J Am Soc Nephrol.

[5] Vieira JM (2007). Effect of acute kidney injury on weaning from mechanical ventilation in critically ill patients. Crit Care Med.

[6] Liu KD, Matthay MA (2008). Advances in critical care for the nephrologist: acute lung injury/ARDS. Clin J Am Soc Nephrol.

[7] Singbartl K (2011). Differential effects of kidney-lung cross-talk during acute kidney injury and bacterial pneumonia. Kidney Int.

[8] Hoag JB (2008). Effects of acid aspiration-induced acute lung injury on kidney function. Am J Physiol Renal Physiol.

[9] Ko GJ, Rabb H, Hassoun HT (2009). Kidney-lung crosstalk in the critically ill patient. Blood Purif.

[10] Basu RK, Wheeler DS (2013). Kidney-lung cross-talk and acute kidney injury. Pediatr Nephrol.

[11] Seaton BA (2010). Review: Structural determinants of pattern recognition by lung collectins. Innate Immun.

[12] Wright JR (2005). Immunoregulatory functions of surfactant proteins. Nat Rev Immunol.

[13] Crouch EC (2000). Surfactant protein-D and pulmonary host defense. Respir Res.

[14] LeVine AM (2000). Distinct effects of surfactant protein A or D deficiency during bacterial infection on the lung. J Immunol.

[15] Giannoni E, Sawa T, Allen L, Wiener-Kronish J, Hawgood S (2006). Surfactant proteins A and D enhance pulmonary clearance of Pseudomonas aeruginosa. Am J Respir Cell Mol Biol.

[16] Madan T (2010). Susceptibility of mice genetically deficient in SP-A or SP-D gene to invasive pulmonary aspergillosis. Mol Immunol.

[17] King BA, Kingma PS (2011). Surfactant protein D deficiency increases lung injury during endotoxemia. Am J Respir Cell Mol Biol.

[18] Waters P, Vaid M, Kishore U, Madan T (2009). Lung surfactant proteins A and D as pattern recognition proteins. Adv Exp Med Biol.

[19] Vandivier RW (2002). Role of surfactant proteins A, D, and C1q in the clearance of apoptotic cells *in vivo* and *in vitro*: calreticulin and CD91 as a common collectin receptor complex. J Immunol.

[20] Atochina-Vasserman EN (2012). S-nitrosylation of surfactant protein D as a modulator of pulmonary inflammation. Biochim Biophys Acta.

[21] Yoshida M, Korfhagen TR, Whitsett JA (2001). Surfactant protein D regulates NF-kappa B and matrix metalloproteinase production in alveolar macrophages via oxidant-sensitive pathways. J Immunol.

[22] Du X (2016). Surfactant Proteins SP-A and SP-D Ameliorate Pneumonia Severity and Intestinal Injury in a Murine Model of Staphylococcus Aureus Pneumonia. Shock.

[23] Madsen J (2000). Localization of lung surfactant protein D on mucosal surfaces in human tissues. J Immunol.

[24] Bourbon JR, Chailley-Heu B (2001). Surfactant proteins in the digestive tract, mesentery, and other organs: evolutionary significance. Comp Biochem Physiol A Mol Integr Physiol.

[25] Stahlman MT, Gray ME, Hull WM, Whitsett JA (2002). Immunolocalization of surfactant protein-D (SP-D) in human fetal, newborn, and adult tissues. J Histochem Cytochem.

[26] Hu F, Liang W, Ren Z, Wang G, Ding G (2012). Surfactant protein D inhibits lipopolysaccharide-induced monocyte chemoattractant protein-1 expression in human renal tubular epithelial cells: implication for tubulointerstitial fibrosis. Clin Exp Immunol.

[27] Liu J (2015). Role of surfactant proteins A and D in sepsis-induced acute kidney injury. Shock.

[28] Hu F (2016). Innate immunity of surfactant proteins A and D in urinary tract infection with uropathogenic Escherichia coli. Innate Immun.

[29] Kurimura Y (2012). Surfactant protein D inhibits adherence of uropathogenic Escherichia coli to the bladder epithelial cells and the bacterium-induced cytotoxicity: a possible function in urinary tract. J Biol Chem.

[30] Knudsen L (2013). Surfactant protein D (SP-D) deficiency is attenuated in humanised mice expressing the Met(11)Thr short nucleotide polymorphism of SP-D: implications for surfactant metabolism in the lung. J Anat.

[31] Hengartner MO (2000). The biochemistry of apoptosis. Nature.

[32] Si MK (2013). Inhibition of poly (adenosine diphosphate-ribose) polymerase attenuates lung-kidney crosstalk induced by intratracheal lipopolysaccharide instillation in rats. Respir Res.

[33] Saito A, Sato H, Iino N, Takeda T (2010). Molecular mechanisms of receptor-mediated endocytosis in the renal proximal tubular epithelium. J Biomed Biotechnol.

[34] Uchino S (2005). Acute renal failure in critically ill patients: a multinational, multicenter study. JAMA.

[35] Angus DC (2001). Epidemiology of severe sepsis in the United States: analysis of incidence, outcome, and associated costs of care. Crit Care Med.

[36] Ranieri VM, Giunta F, Suter PM, Slutsky AS (2000). Mechanical ventilation as a mediator of multisystem organ failure in acute respiratory distress syndrome. JAMA.

[37] Kuiper JW, Groeneveld AB, Slutsky AS, Plotz FB (2005). Mechanical ventilation and acute renal failure. Crit Care Med.

[38] Ventilation with lower tidal volumes as compared with traditional tidal volumes for acute lung injury and the acute respiratory distress syndrome. The Acute Respiratory Distress Syndrome Network. *N Engl J Med* **342**, 1301–1308, 10.1056/NEJM200005043421801 (2000). 10.1056/NEJM200005043421801 10793162

[39] Haczku A (2008). Protective role of the lung collectins surfactant protein A and surfactant protein D in airway inflammation. J Allergy Clin Immunol.

[40] Calfee CS (2015). Distinct molecular phenotypes of direct vs indirect ARDS in single-center and multicenter studies. Chest.

[41] Chertow GM, Burdick E, Honour M, Bonventre JV, Bates DW (2005). Acute kidney injury, mortality, length of stay, and costs in hospitalized patients. J Am Soc Nephrol.

[42] Havasi A, Borkan SC (2011). Apoptosis and acute kidney injury. Kidney Int.

[43] Imai Y (2003). Injurious mechanical ventilation and end-organ epithelial cell apoptosis and organ dysfunction in an experimental model of acute respiratory distress syndrome. JAMA.

[44] Fadok VA (2000). A receptor for phosphatidylserine-specific clearance of apoptotic cells. Nature.

[45] Clark H (2002). Surfactant protein D reduces alveolar macrophage apoptosis *in vivo*. J Immunol.

[46] Liu Z (2015). Innate Immune Molecule Surfactant Protein D Attenuates Sepsis-induced Acute Pancreatic Injury through Modulating Apoptosis and NF-kappaB-mediated Inflammation. Sci Rep.

[47] Djiadeu P (2017). Surfactant protein D regulates caspase-8-mediated cascade of the intrinsic pathway of apoptosis while promoting bleb formation. Mol Immunol.

[48] Djiadeu P, Kotra LP, Sweezey N, Palaniyar N (2017). Surfactant protein D delays Fas- and TRAIL-mediated extrinsic pathway of apoptosis in T cells. Apoptosis.

[49] Ghosh S, Hayden MS (2012). Celebrating 25 years of NF-kappaB research. Immunol Rev.

[50] Guijarro C, Egido J (2001). Transcription factor-kappa B (NF-kappa B) and renal disease. Kidney Int.

[51] Marko L (2016). Tubular Epithelial NF-kappaB Activity Regulates Ischemic AKI. J Am Soc Nephrol.

[52] Gardai SJ (2003). By binding SIRPalpha or calreticulin/CD91, lung collectins act as dual function surveillance molecules to suppress or enhance inflammation. Cell.

[53] Janssen WJ (2008). Surfactant proteins A and D suppress alveolar macrophage phagocytosis via interaction with SIRP alpha. Am J Respir Crit Care Med.

[54] Yamazoe M (2008). Pulmonary surfactant protein D inhibits lipopolysaccharide (LPS)-induced inflammatory cell responses by altering LPS binding to its receptors. J Biol Chem.

[55] Zhang Z, Abdel-Razek O, Hawgood S, Wang G (2015). Protective Role of Surfactant Protein D in Ocular Staphylococcus aureus Infection. PLoS One.

[56] Glasser SW, Burhans MS, Eszterhas SK, Bruno MD, Korfhagen TR (2000). Human SP-C gene sequences that confer lung epithelium-specific expression in transgenic mice. Am J Physiol Lung Cell Mol Physiol.

[57] Fisher JH (2000). Pulmonary-specific expression of SP-D corrects pulmonary lipid accumulation in SP-D gene-targeted mice. Am J Physiol Lung Cell Mol Physiol.

[58] Wang G, Guo X, Diangelo S, Thomas NJ, Floros J (2010). Humanized SFTPA1 and SFTPA2 transgenic mice reveal functional divergence of SP-A1 and SP-A2: formation of tubular myelin *in vivo* requires both gene products. J Biol Chem.

[59] Ge L (2016). Differential susceptibility of transgenic mice expressing human surfactant protein B genetic variants to Pseudomonas aeruginosa induced pneumonia. Biochem Biophys Res Commun.

[60] Xu Y (2016). Differential Susceptibility of Human Sp-B Genetic Variants on Lung Injury Caused by Bacterial Pneumonia and the Effect of a Chemically Modified Curcumin. Shock.

[61] Matute-Bello G (2011). An official American Thoracic Society workshop report: features and measurements of experimental acute lung injury in animals. Am J Respir Cell Mol Biol.

[62] Melnikov VY (2001). Impaired IL-18 processing protects caspase-1-deficient mice from ischemic acute renal failure. J Clin Invest.

[63] Wang G, Christensen ND, Wigdahl B, Guttentag SH, Floros J (2003). Differences in N-linked glycosylation between human surfactant protein-B variants of the C or T allele at the single-nucleotide polymorphism at position 1580: implications for disease. Biochem J.

[64] Terryn S (2007). A primary culture of mouse proximal tubular cells, established on collagen-coated membranes. Am J Physiol Renal Physiol.

[65] Chen WC, Lin HH, Tang MJ (2014). Regulation of proximal tubular cell differentiation and proliferation in primary culture by matrix stiffness and ECM components. Am J Physiol Renal Physiol.

[66] Kelley R (2010). Tubular cell-enriched subpopulation of primary renal cells improves survival and augments kidney function in rodent model of chronic kidney disease. Am J Physiol Renal Physiol.

[67] Baer PC, Nockher WA, Haase W, Scherberich JE (1997). Isolation of proximal and distal tubule cells from human kidney by immunomagnetic separation. Technical note. Kidney Int.

